# The value of the hedgehog signal in osteoblasts in fluoride-induced bone-tissue injury

**DOI:** 10.1186/s13018-021-02287-8

**Published:** 2021-02-26

**Authors:** Chaonan Deng, Lin Xu, Ying Zhang, Lina Zhao, Yan Linghu, Yanni Yu

**Affiliations:** 1 Department of Pathology, Affiliated Hospital of GuiZhou Medical University, No. 28 of Guiyi Street, Guiyang, 550004 China; 2 Department of Pathology, GuiZhou Medical University, Guiyang, 550004 China; 3 Guiyang Maternal and Child Care Hospital, 550004 Guiyang, China

**Keywords:** Osteoblasts, Hh signal, Fluorosis, Bone-tissue injury, Skeletal fluorosis

## Abstract

**Objective:**

This study was designed to observe the expression of important hedgehog (Hh) signal factors in the bone tissue of rats with chronic fluorosis and cultured osteoblasts in order to investigate the role and significance of the Hh signal in fluoride-induced bone injury.

**Methods:**

Healthy Sprague-Dawley (SD) rats were randomly divided into four groups: the control group, the fluorosis group (F Group), the fluoride + blocker group (F + Cycl group: rats were treated with fluoride + cyclopamine), and the fluoride + blocker control group (F + DMSO group). After 6 months of intervention, the urinary fluoride content of rats in each group was detected. The primary osteoblasts of rats were selected for cell experiment, and the experiment was carried out after the cells were passaged from the second to the fourth generation.

**Results:**

The proliferation rate of primary rat osteoblasts presented time-affected and dose-affected relationships in a short time under treatment with a low dose of sodium fluoride (NaF), but the proliferation of osteoblasts was inhibited by long-term and high-dose NaF exposure. In the F group, the alkaline phosphatase (ALP) activity of osteoblasts increased gradually. The ALP activity was lower in the F + Cycl group than in the F group, and there was no significant difference between the F + DMSO group and F group. With the increase in fluoride exposure, the expression of Hh signal factors and osteogenic-related factor proteins increased gradually. The expressions of Indian hedgehog (Ihh), smoothened (Smo), Glioma-associated oncogene homolog (Gli) 2, and Runt-related transcription factor 2 (Runx2)in the F + Cycl group increased with the dose of fluoride but they were significantly inhibited compared with the F group. Compared with the control group, the content of urinary fluoride in the F group was significantly higher (*P* < 0.05), but there was no significant change in urinary fluoride content in the F + Cycl group and the F + DMSO group. Compared with the control group, the serum bone alkaline phosphatase (BALP) contents of rats in the other groups increased after 6 months’ intake of fluoride water (*P* < 0.05). After drug blocking, the serum BALP content in the F + Cycl group was lower than that in the F + DMSO group (*P* < 0.05). The BALP content in the F + DMSO group was similar to that in the F group: it did not decrease. The mRNA expressions of Ihh, Smo, Gli2, and Runx2 in bone tissue of the F group were significantly higher than those in the control group (*P* < 0.05). After cyclopamine blocking, the expressions decreased (*P* < 0.05), but the differences between the F + DMSO group and F group were not statistically significant.

**Conclusion:**

Hh signal plays an important role in fluoride-induced bone injury. The effective inhibition of cyclopamine is expected to be a new target for the treatment of skeletal damage caused by fluorosis.

## Introduction

Endemic fluorosis is a systemic, chronic, toxic disease characterized by skeletal fluorosis and dental fluorosis, which is caused by the long-term intake of fluorine by residents through drinking water, food, air, and other means that exceeds normal physiological needs. It is one of the most widespread and serious endemic diseases in China [1]. After the implementation of prevention and control measures, such as improvements to the water supply and to stoves, the prevalence of endemic fluorosis has been partially controlled. However, due to the complexity of the natural or ecological environment, the pathogenesis of endemic fluorosis is not very clear [2, 3]. Mak et al. found that selective upregulation of the Hh signal in mature osteoblasts could lead to significant increases in osteogenesis and bone resorption [4]; this is very similar to the pathology of skeletal fluorosis observed in many previous studies. Therefore, we speculate that the hedgehog (Hh) signal may affect the balance of bone remodeling by participating in the differentiation, proliferation, and apoptosis of osteoblasts and accordingly play an important role in the occurrence and development of skeletal fluorosis.

This study was designed to observe the expression of important Hh signal factors in the bone tissue of rats with chronic fluorosis and cultured osteoblasts in order to investigate the role and significance of the Hh signal in fluoride-induced bone injury.

## Materials and methods

### Cell culture

The primary osteoblasts of rats were taken from the skulls of specific pathogen-free (SPF) Sprague-Dawley (SD) rats (provided by the Animal Experimental Center of Guizhou Medical University) aged 24–48 h. These were used for cell experiments after being passaged from the second to the fourth generation.

### The experimental group

The present study meets the requirements of the Declaration of Helsinki of the World Medical Association and has been approved by the Ethics Committee of our hospital. In this study, 48 healthy SD rats (100–120 g) (Medical Laboratory Animal Center, Daping Hospital, the Third Military Medical University, Certificate No. SCXK [Yu] 2007-0005) were used. These rats were randomly divided into four groups: the control group, the fluorosis group (F Group), the fluoride + blocker group (F + Cycl group: rats were treated with fluoride + cyclopamine), and the fluoride + blocker control group (F + dimethyl sulfoxide [DMSO] group). There were 12 rats in each group; half were males and the half were females in each group. All groups of rats were fed with standard solid feed (fluorine content less than 1 mg/kg) prepared by the Animal Center of Guizhou Medical University. The rats in the control group were given tap water (fluorine content < 1 ppm). In the F group, the tap water had 50 ppm of sodium fluoride (NaF) added. In the F + Cycl group, after 6 months of feeding with tap water containing 50 ppm of NaF, the rats were injected intraperitoneally with 10 mg/kg of cyclopamine while continuing the fluoride intake, q.o.d., three times in total. In the F + DMSO group, after 6 months of feeding with tap water containing 50 ppm of NaF, the rats were injected intraperitoneally with 10 mg/kg of DMSO while continuing the fluoride intake, q.o.d., three times in total. The urinary fluoride content of the rats in each group was detected. Some epiphyseal tissues from the femoral shaft were fixed with neutral formalin and decalcified with ethylenediaminetetraacetic acid (EDTA). The rest of the bone tissues were preserved at – 80 °C.

### Main reagents and instruments

Cyclopamine (Selleck, USA); rat serum ELISA Kit (bone alkaline phosphatase [BALP], R&D, USA); rabbit anti-mouse polyclonal antibodies Ihh, Smo, Gli2, and Runx2 (Santa Cruz, USA); diaminobiphenyl amine (DAB, Guangzhou Ascend Biotechnology Co., Ltd., China); enzyme micro-plate reader (Bio-Rad, USA); real-time polymerase chain reaction (PCR) machine (Applied Biosystems, USA).

### Experimental methods

#### Culture and identification of rat primary osteoblasts

Osteoblasts from the calvaria of the newborn rats were isolated by secondary enzyme digestion and cultured, and the purity of the osteoblasts was determined by alkaline phosphatase staining.

#### Determining the use concentration of NaF and cyclopamine

After 72 h of cell subculture, different amounts of NaF solution were added into the cell culture medium to make final concentrations of NaF solution of 0 mg/L, 2.5 mg/L, 5 mg/L, 10 mg/L, and 20 mg/L, respectively. Then the cells were cultured at different concentrations for 24 h, 48 h, and 72 h. The CCK8 method was used to detect the cell proliferation rate to determine the suitable concentration of fluoride.

The proliferation rate of the cultured cells was measured after 24 h, 48 h, and 72 h of treatment with cyclopamine (the final concentration was 0 μmol/L, 5 μmol/L, 10 μmol/L, and 20 μmol/L) to determine the best dosage.

#### Detection of the urine’s fluorine content

The 24 h urine of all rats was collected and the fluoride content in the urine was detected by the fluoride ion selective electrode method.

#### Determination of the serum bone alkaline phosphatase

Blood was sampled from the femoral arteries of the rats, centrifuged, and the upper serum was used to determine the BALP level, following the steps in the instructions of the kit.

#### Bone histopathology

The epiphysis of the femoral shaft of the rats was obtained, fixed with 4% neutral formaldehyde, decalcified with EDTA, embedded with paraffin, and then underwent hematoxylin and eosin (H&E) staining. The trabecular width, trabecular density, and cortical thickness were measured at 1 mm below the epiphyseal line of the femoral metaphysis.

#### Detection of the mRNA of the Hh pathway and osteogenic-related factors

The total RNA of the rat bone tissue and the cultured osteoblasts was extracted and reversely transcribed into cDNA using random primers. A real-time PCR of the gene to be tested was carried out. The primers were designed and synthesized by Shanghai GeneCore Bioengineering Co., Ltd. Reaction conditions: pre-denaturation at 95 °C for 10 min, with the reaction terminated after 40 cycles at 95 °C for 15 s and at 60 °C for 30 s. At the end of the reaction, the software automatically generated the Ct value, and 2^−△Ct^ was calculated for comparison. The primer sequences were as follows: Runx2: forward primer 5-ACAGCACCTTCAGCACTCT-3, reverse primer 5-AAGTTCTTGGCTATTACGACA-3.

#### Detection of the protein of the Hh pathway and osteogenic-related factors

Immunohistochemistry (IHC) and western blot were used to observe the location and expression of the Hh pathway and osteogenic-related factors.

##### IHC

The sections were dewaxed, incubated with 3% H_2_O_2_ for 20 min, repaired with high pressure for 2.5 min, naturally cooled down to room temperature, and incubated with blocking buffer for 25 min. Then, the blocking buffer was discarded without washing, and the sections were dropped with diluted primary antibody and left standing at 4 °C overnight. The next day, the sections were taken out and rewarmed for 5 min, a second antibody was added for 25 min then horseradish peroxidase was added for 25 min. The sections were colored with DAB, then the reaction was terminated with flow water and the sections were counterstained with hematoxylin, dehydrated, treated with xylene until they became transparent, and sealed.

##### Western blot

The total RNA of the rat bone tissue and cultured osteoblasts was extracted, lysated on ice with a radio-immunoprecipitation assay (RIPA) lysis buffer, and phenylmethylsulfonyl fluoride (PMSF) for 1 h, and centrifuged at 2500 rpm and 4 °C for 30 min. Then the protein concentration was determined by the BCA method. Equal amounts of total protein (40 μg) of protein were taken to undergo polyacrylamide gel electrophoresis (PAGE), electro-transferred to a polyvinylidene fluoride (PVDF) membrane, and blocked with 1 × Tris buffered saline/Tween (TBS-T) solution containing 5% bovine serum albumin (BSA) for 1 h. Then, the first antibodies, Ihh (1:200), Smo (1:200), Gli2 (1:500), and Runx2 (1:500) were added, and the samples were incubated at 4 °C overnight. The second antibody (1:3000) was then added and the samples were incubated at room temperature for 1 h the next day, exposed and developed, and Image J gray analysis software was used for quantitative analysis.

### Statistical analysis

Data were analyzed using the statistical software SPSS 20.0. Measurement data were expressed as the mean ± standard deviation (‾x ± SD), and count data were expressed as percentages (%). Comparisons between multiple groups were conducted using univariate analysis of variance, and a post hoc test was conducted using the least significant difference (LSD). Abnormally distributed measurement data were compared among groups using a nonparametric test. Count data were evaluated using the chi-square test. *P* < 0.05 was considered statistically significant.

## Results

### Culture and identification of rat primary osteoblasts

**Fig. 1:**
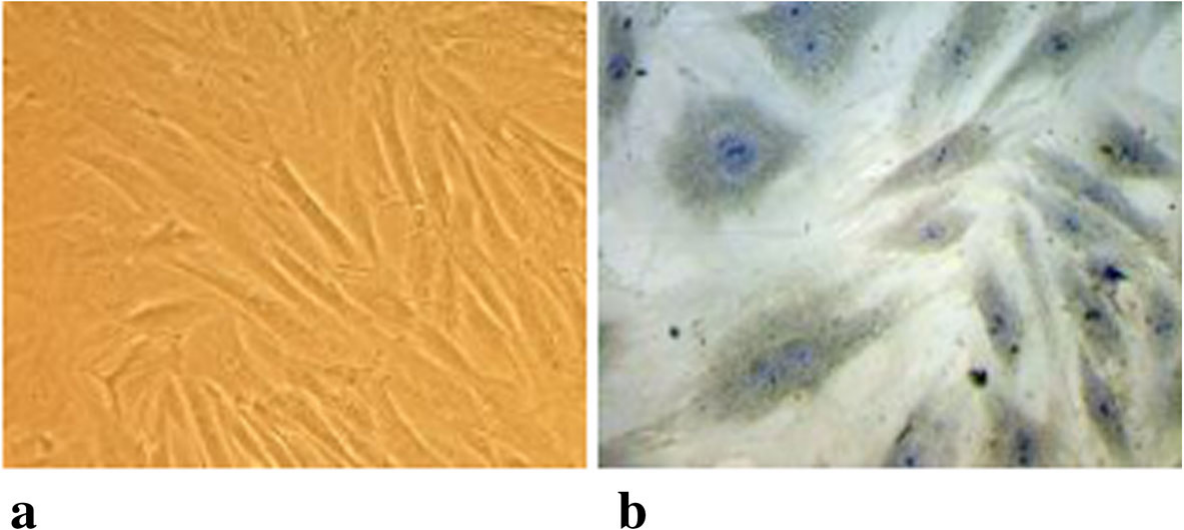
Primary rat osteoblasts. **a** Primary rat osteoblasts, observed in the inverted position, × 200. **b** Osteoblasts are stained with alkaline phosphatase (× 400)

The primary rat osteoblasts were obtained by secondary enzyme digestion and passaged to the second generation. Osteoblasts were spindle-shaped, fusiform or triangular, with protuberance. The nuclei were elliptic. The cells were adherent to the wall, and the refraction was good. Alkaline phosphatase staining showed that there were gray-black and gray-brown deep-staining particles in the cytoplasm (Fig. 1).

### Time- and dose-effect relationships between the osteoblast proliferation rate and NaF treatment and the screening of cyclopamine concentration

**Fig. 2:**
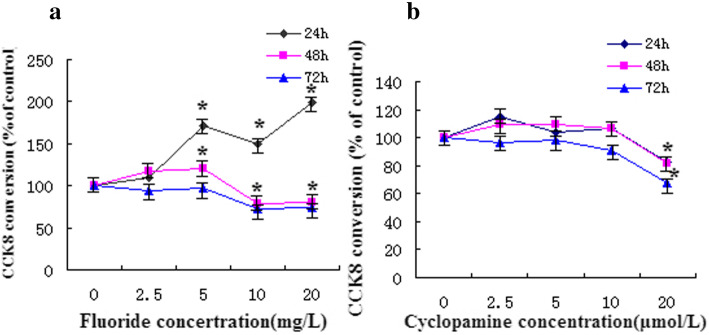
The rate of cell proliferation treated with NaF or Cycl. **a** The rate of cell proliferation treated with NaF. **b** The rate of cell proliferation treated with Cycl

As shown by Fig. 2a, osteoblasts were treated with different concentrations of NaF for 24 h, 48 h, and 72 h. The result revealed that with the increase of fluoride dose and time, the effects of fluoride on osteoblast proliferation were different. At 24 h of treatment, the proliferation rate of osteoblasts was gradually increased; at 48 h of treatment, the proliferation rate of cells treated with 2.5 mg/L and 5 mg/L NaF was increased, but the proliferation rate of cells treated with 10 mg/L and 20 mg/L NaF was rapidly decreased; at 72 h of treatment, the proliferation rate of cells treated with various concentrations of NaF was decreased.

As shown by Fig. 2b, osteoblasts were treated with different concentrations of cyclopamine for 24 h, 48 h, and 72 h to select the appropriate treatment concentration of cyclopamine. The results revealed that with an increase in cyclopamine concentration (2.5 μmol/L, 5 μmol/L), the cell proliferation rate was decreased gradually. When cyclopamine concentration was > 10 μmol/L, the cell viability was significantly affected at the three time points.

The purpose of this study was to investigate the mechanism of osteoblast proliferation induced by fluoride. Therefore, in the subsequent experiment, the cells were treated with 2.5 mg/L and 5 mg/L NaF for 48 h and then treated with 5 μmol/L cyclopamine for 48 h.

### Detection of alkaline phosphatase activity in cultured osteoblasts

**Fig. 3:**
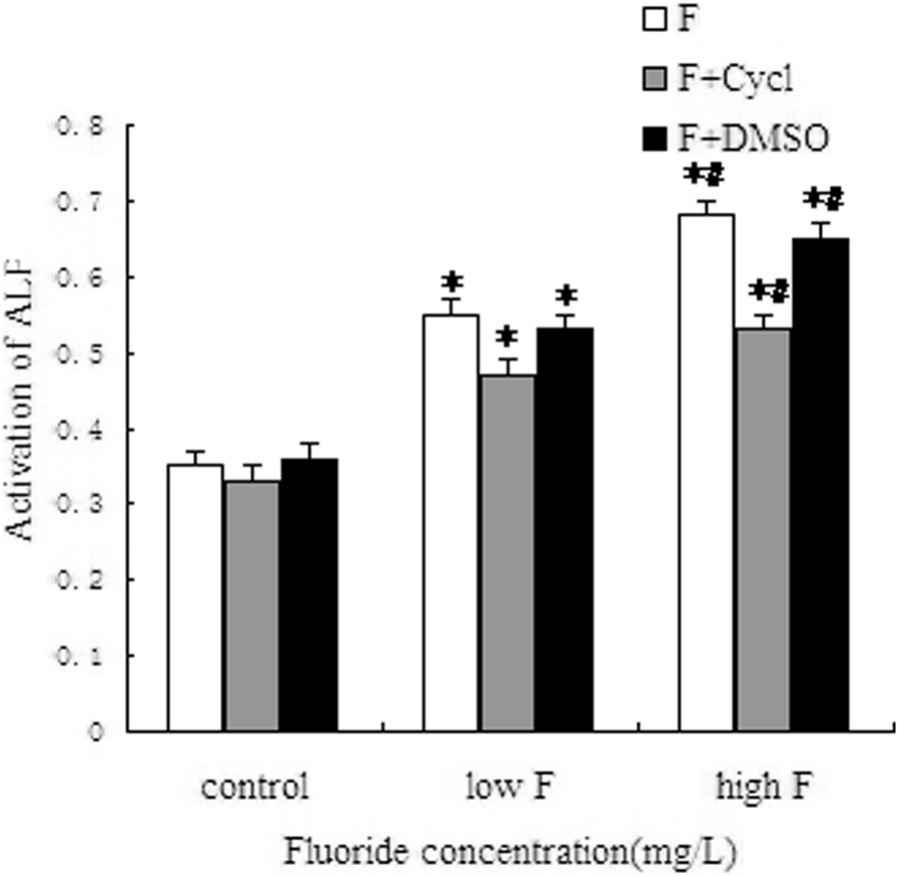
The effect of ALP activity of osteoblasts treated with fluoride exposure using Cycl. *Compared with the control group *P* < 0.05. #Compared with the low fluoride group *P* < 0.05

In the present study, after screening drug concentration, cells were treated with 2.5 mg/L and 5 mg/L NaF for 48 h and then treated with 5 μmol/L cyclopamine for 48 h. Then ALP activity in osteoblasts was detected. The results revealed that in the F group, the ALP activity of osteoblasts was gradually increased; the change trend was basically the same as that of the cell proliferation rate. The ALP activity was lower in the F + Cycl group than in the F group, and there was no significant difference between the F + DMSO group and the F group (Fig. 3).

### MRNA expressions of the Hh signaling pathway and osteogenic-related factors in cultured osteoblasts

**Fig. 4:**
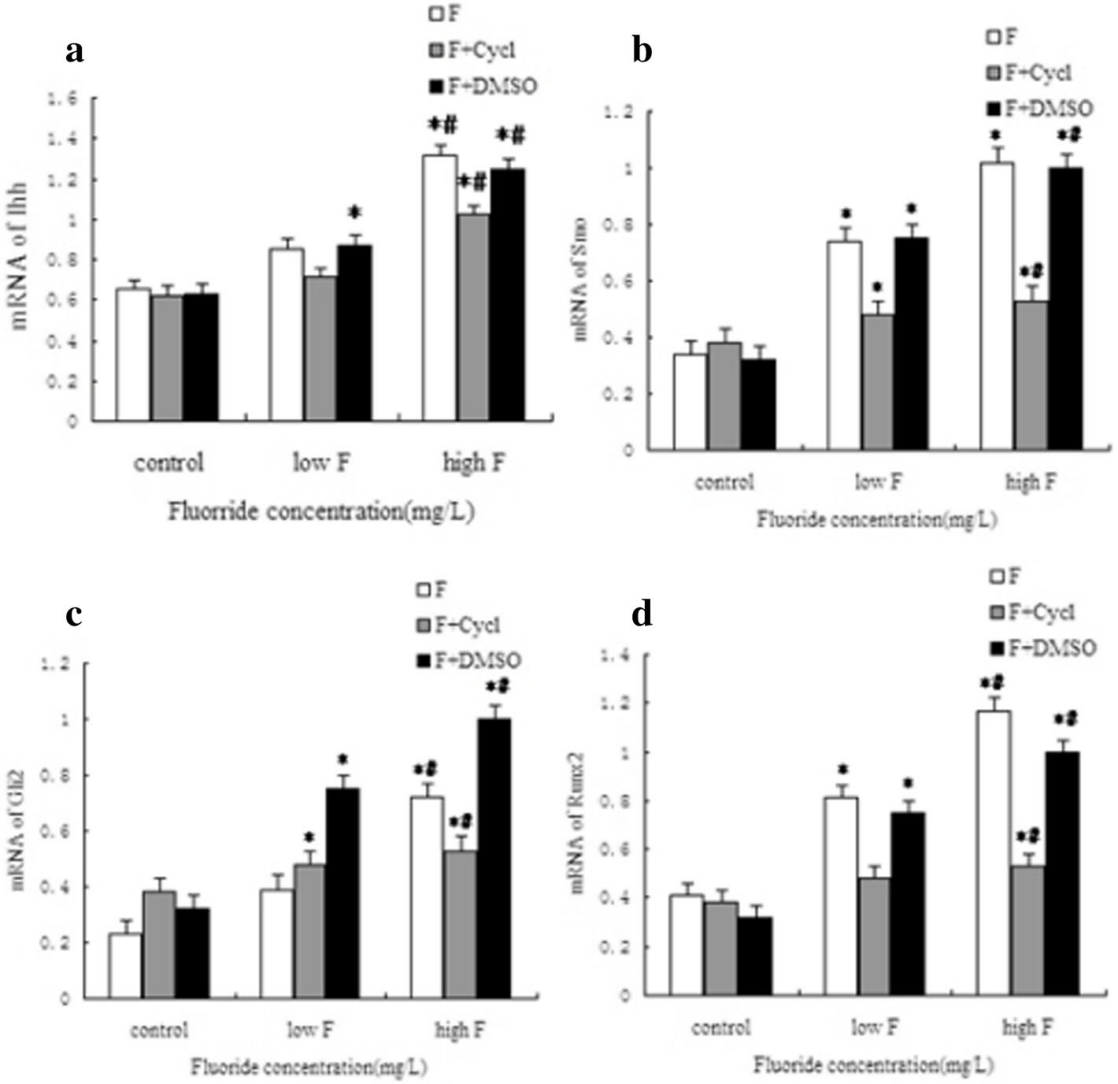
The mRNA expressions of Ihh, Smo, Gli2, and Runx2 in osteoblasts treated with fluoride exposure influenced by Cycl. **a**–**d** Exhibit the mRNA expressions of Ihh, Smo, Gli2, and Runx2, respectively, in cultured osteoblasts. *Compared with the control group *P* < 0.05. #Compared with the low fluoride group *P* < 0.05

As shown by Fig. 4, with the increase in fluoride exposure dose, the expressions of Ihh, Smo, Gli2, and Runx2 in osteoblasts were increased gradually. The differences were statistically significant compared with the control group. Compared with the control group, in the F + Cycl group, the expressions of all indexes were increased in the low-dose group and the dose group after treatment, but the expressions were lower than that of the F sub-group with corresponding doses. There were no significant differences between the F + DMSO group and the F group.

### Protein expressions of the Hh signaling pathway and osteogenic-related factors in cultured osteoblasts

**Fig. 5:**
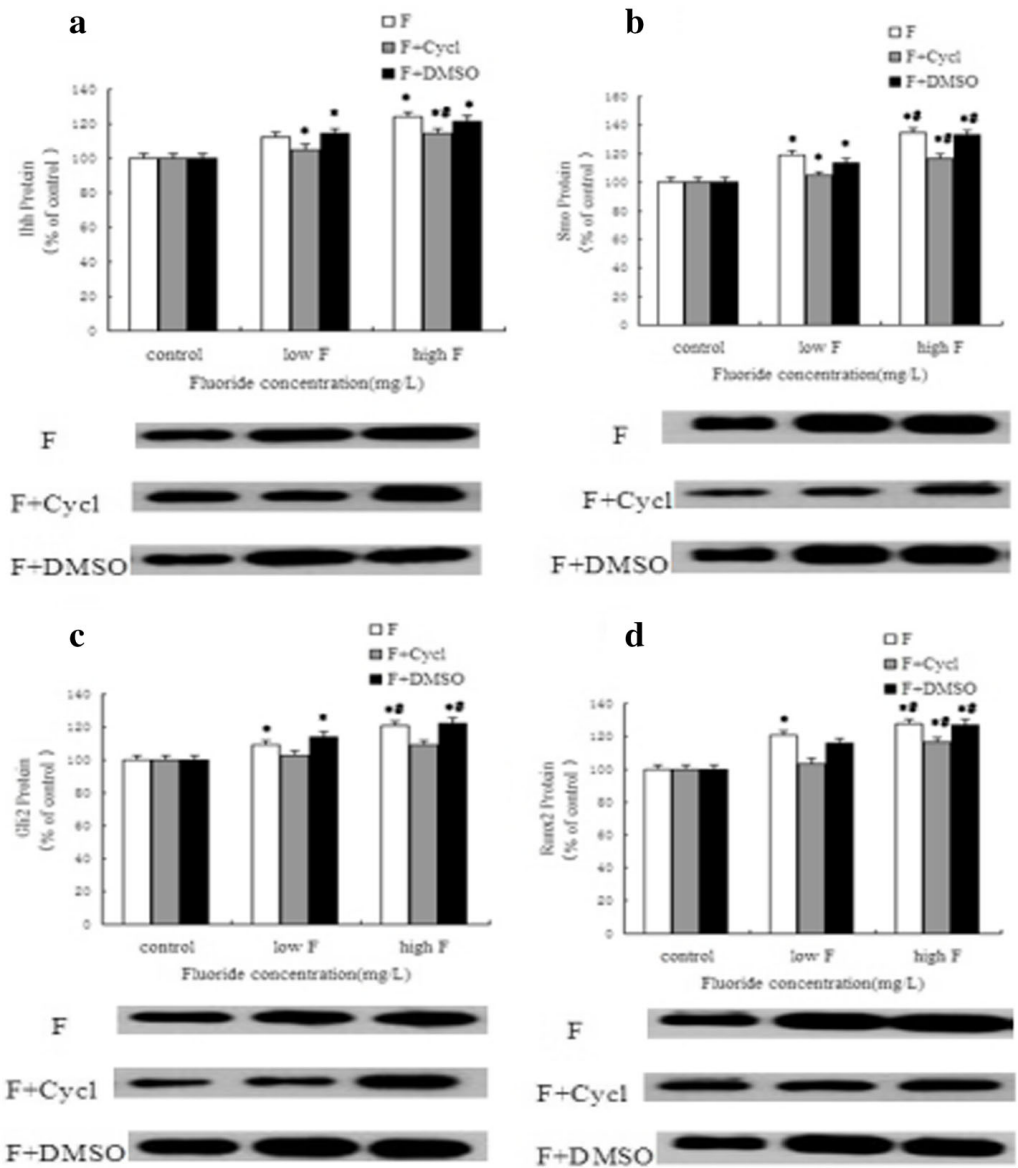
The protein expressions of Ihh, Smo, Gli2, and Runx2 in osteoblasts treated with fluoride exposure using Cycl. **a**–**d** exhibit the protein expressions of Ihh, Smo, Gli2 and Runx2, respectively, in cultured osteoblasts. *Compared with the control group *P* < 0.05. #Compared with the low fluoride group *P* < 0.05

As shown by Fig. 5, the protein expressions of all indexes were basically the same as the mRNA expressions of corresponding indexes. With the increase in fluoride exposure dose, the protein expressions of the Hh signal factors and osteogenic-related factors were gradually increased. The expressions of Ihh, Smo, Gli2, and Runx2 in the F + Cycl group were increased with the dose of fluoride but they were significantly inhibited compared with the F group. There were no significant differences in the protein expressions of all indexes between the F + DMSO group and the F group.

### Urinary fluoride content and serum bone alkaline phosphatase content in rats

Compared with the control group, the urinary fluoride content in the F group was significantly higher (*P* < 0.05), but there was no significant change in urinary fluoride content in the F + Cycl group and the F + DMSO group. Compared with the control group, the serum BALP contents of rats in the other groups were increased after 6 months’ intake of fluoride water (*P* < 0.05). After drug blocking, the serum BALP content in the F + Cycl group was lower than that in the F + DMSO group (*P* < 0.05). The serum BALP content in the F + DMSO group was similar to that in the F group: it did not decrease (Table 1).

**Table 1:** Urine fluorine, serum BALP content and bone tissue Ihh, Shh, Ptch1, and Smo mRNA expression in each group

Groups	The number of cases	The urinary fluoride content(mg/L)	BALP(IU/L)	Ihh	Smo	Gli2	Runx2
Control group	12	1.36 ± 0.51	27.78 ± 4.09	0.73 ± 0.19	0.14 ± 0.04	0.83 ± 0.19	0.37 ± 0.10
F group	12	7.60 ± 0.61^*^	57.46 ± 3.99^*^	1.39 ± 0.36^*^	0.40 ± 0.15^*^	1.40 ± 0.31^*^	0.57 ± 0.19^*^
F + Cycl group	12	7.55 ± 0.32^*^	53.55 ± 2.22^*#&^	0.81 ± 0.28^#&^	0.16 ± 0.03^#&^	0.75 ± 0.17^#&^	0.42 ± 0.09^#&^
F + DMSO group	12	7.55 ± 0.19^*^	57.56 ± 4.23^*^	1.31 ± 0.34^*^	0.39 ± 0.10^*^	1.26 ± 0.35^*^	0.55 ± 0.17^*^

*Compared with control group, *P* < 0.05

^#^Compared with F group, *P* < 0.05

^&^Compared with F + DMSO group, *P* < 0.05

### MRNA expressions of the Hh signaling pathway and osteogenic-related factors in bone tissue

The mRNA expressions of Ihh, Smo, Gli2, and Runx2 in the bone tissue of the F group were significantly higher than those in the control group (*P* < 0.05). After cyclopamine blocking, the expressions were decreased (*P* < 0.05), but the differences between the F + DMSO group and F group were not statistically significant (Table 1).

### Protein expressions of the Hh signaling pathway and osteogenic-related factors in bone tissue

**Fig. 6:**
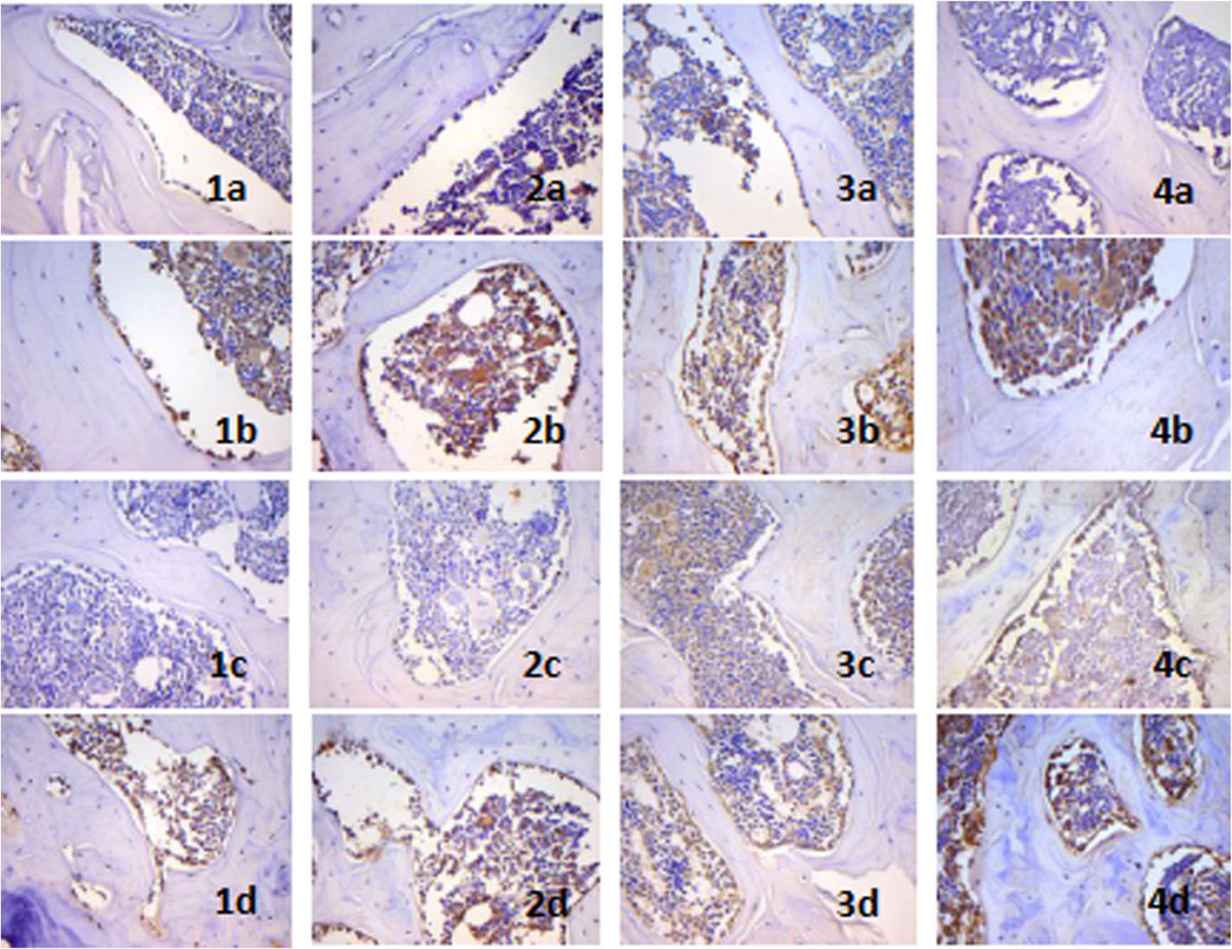
The immunoreactivities of Ihh, Smo, Gli2, and Runx2 on osteoblasts in the experimental rats. **a** Control group. **b** F group. **c** F + Cycl group. **d** F + DMSO group. 1. Ihh, 2. Smo, 3. Gli2, 4. Runx2, SP method (×400)

**Fig. 7:**
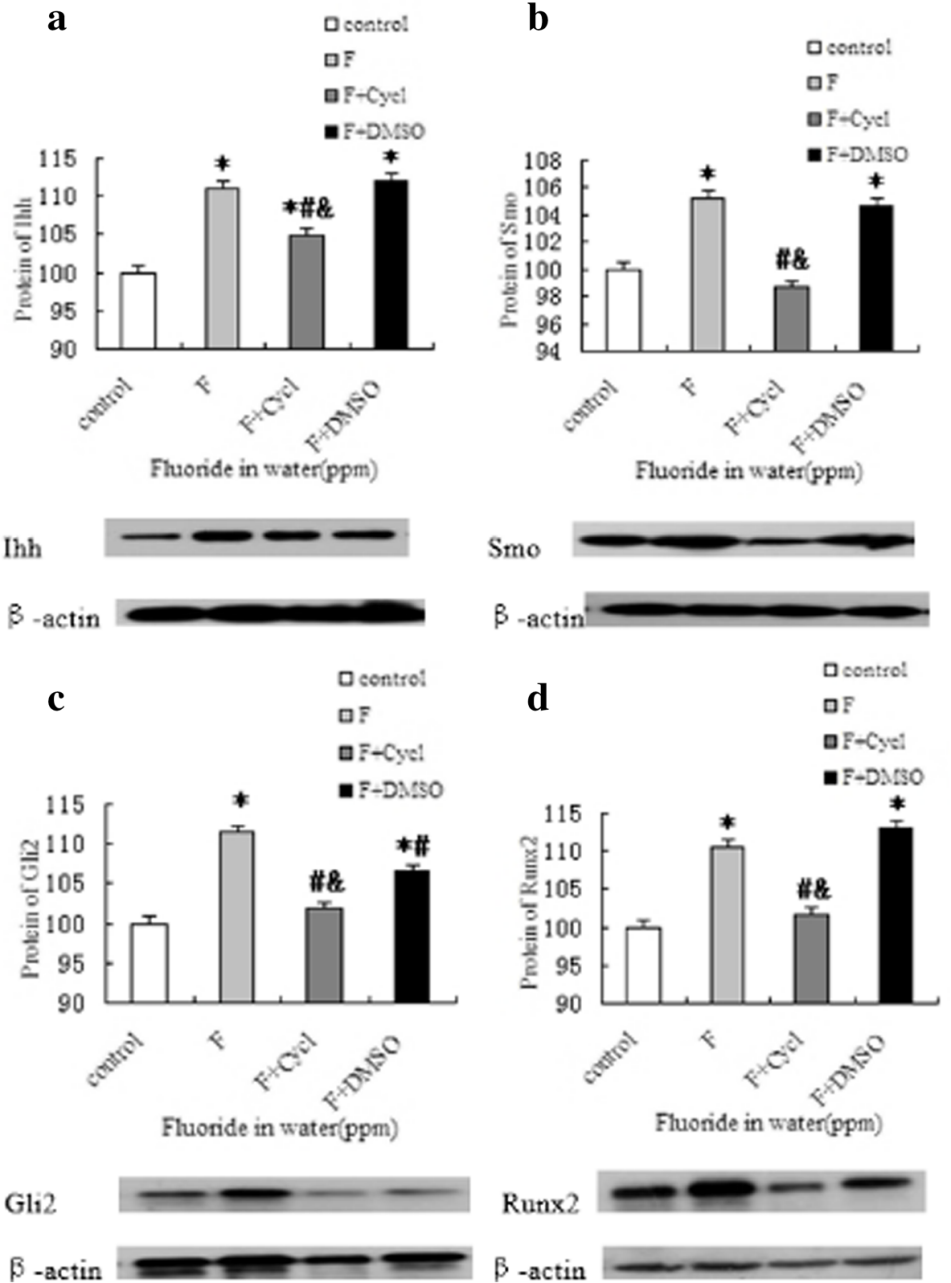
The protein expressions of Ihh, Smo, Gli2, and Runx2 in the experimental rats’ bone. **a**–**d** Exhibit the protein expressions of Ihh, Smo, Gli2, and Runx2, respectively, in bone tissue. *Compared with the control group *P* < 0.05. #Compared with the F group *P* < 0.05, &Compared with the F + DMSO group *P* < 0.05

Immunohistochemistry revealed that Ihh, Smo, Gli2, and Runx2 were all positively expressed in different degrees in osteoblasts of metaphysis, the expressions were all increased after exposure to fluoride, and the expressions were lower in the F + Cycl group than in the F group and F + DMSO group (*P* < 0.05) (Figs. 6 and 7).

## Discussion

In the present study, rats were fed with fluoride water for 6 months. Following this, the fluoride content in their urine was significantly higher than that of the control group. This suggests that the fluorosis model was established successfully. This study revealed that the serum BALP level was significantly higher in the F group than in the control group. After exposure to fluoride, the bone cortex thickened, the bone trabecular thickened, and bone trabecular density increased. Bone formation was obviously enhanced in rats with chronic fluorosis. However, the differences in bone morphometric indexes among the F group, F + Cycl group and F + DMSO group were not statistically significant. The reason may be that changes in serological indexes appear before those in morphology. Thus, it is speculated that fluoride can enhance osteogenesis, and serum BALP can be used as a marker to judge the effect of fluoride on bone tissue.

Hedgehog is a secreted glycoprotein that can bind to the Patched (Ptch) receptor on the cell membrane and activate the effector Gli1 to change the structure of the receptor [5, 6]. Lozito et al. carried out a comparative study on the regenerating tail and non-regenerating tail of geckos [7]. The results confirmed that Ihh was involved in the ossification of the growth plate and participated in the ossification of the growth plates of different parts of the body through different mechanisms; the Ihh signal may increase bone formation [8]. Dysfunction in the Hh signal led to the destruction of bone development and bone balance [9]. In mature osteoblasts, selective upregulation of the Hh signal led to increased bone formation and excessive bone resorption [4]. These results are very similar to the pathological manifestations of osteofluorosis observed in previous studies. The molecules that mediate the bone-repair process in adulthood are Shh and Ihh in the Hh signaling pathway [9].

Cyclopamine, a specific blocker of the Hh signal, is an isosteroidal alkaloid. It can directly act on Smo and inhibit the activity of the Hh signal [10]. The present study was designed based on the methods of Sanchez [11] and Hirotsu et al. [12]. The half-maximal inhibitory concentration (IC_50_) of cyclopamine was 2.5 μM, and 1–10 μM cyclopamine can significantly inhibit the osteogenic differentiation of the mouse mesenchymal stem-cell line, C3H10T1/2. In addition, in the present study, real-time PCR revealed that the relative expressions of Ihh, Gli2, and Smo were increased in osteoblasts exposed to fluoride, and the mRNA and protein expressions of Ihh, Gli2, and Smo were also upregulated in the bone tissue of rats with fluorosis. After blocking with cyclopamine, the mRNA and protein expressions of Ihh, Gli2, and Smo were decreased. Therefore, the expression of Gli reflects the activation of the Hh signal. In the present study, Gli2 expression was significantly increased. This suggests that the Hh signal is activated in osteoblasts after exposure to fluoride. The investigators consider that after this signal is activated, excessive ligand Hh binds to Ptch, Smo is freed, and Smo promotes its activation factor Gli to enter the nucleus through the action of proteolytic enzyme, to enhance the transcription of the Hh signal target gene. The Ihh signal plays a critical role in the mineralization of the tendon end of fibrocartilage [13]. After blocking with cyclopamine, the decrease in osteoblast proliferation and differentiation ability and the decrease of serum BALP level can be partly attributed to the inhibition of Ihh expression.

There is a broad connection between the Hh signal and other signaling pathways. For example, knockdown of Ihh can inhibit the growth and differentiation of chondrocytes. The reason may be that it is closely related to the transforming growth factor—β(TGF-β)/Smad and osteoprotegerin/receptor activator ofNF-κB ligand (OPG/RANKL) signaling pathways [14]. The results of previous studies revealed that exendin-4 can enhance ALP activation and mineralization nodule formation of osteoblasts, and can also promote the differentiation of osteoblast precursor cells into osteoblasts by upregulating the expressions of glucagon-like peptide-1 receptor, Hh, Gli1, Runx2, and osteocalein through the Hh/Gli1 pathway [15, 16]. Runx2 can be expressed in undifferentiated mesenchymal stem cells and can directly regulate the expressions of Ihh, Gli1, and Patched-1(Ptc1) [17]. The results of this experiment revealed that after exposure to fluorine, the expressions of mRNA and the Runx2 protein were the same as those of the Hh signal factor. This suggests that in the process of osteoblast proliferation, Runx2 and Hh signals may regulate each other and induce osteoblast differentiation [18]. Fluoride can increase the protein and mRNA expressions of SHH, SMO, and GLI1 in liver cells of chronic fluorosis rats, and may be inhibited by cyclopamine. The SHH signaling pathway plays an important role in the pathogenesis of liver caused by fluorosis [19]. The analysis of the in vivo experiment revealed that overexpression of Gli1 could upregulate early osteogenesis-related factors; Gli1 could induce early osteogenic differentiation in a Runx2-dependent manner to a certain extent. In the absence of Runx2, overexpression of Ihh or Gli2 could not enhance the activity of ALP. Runx2 is a molecular marker of osteoblast proliferation and early differentiation. These results further confirm that the Hh signal is mainly responsible for the proliferation and differentiation of osteoblasts. The investigators consider that under the action of fluorine, the process is mainly that Ihh activates Gli2 to transcribe the signal into the nucleus to induce Runx2 expression through the mediation of Smo. In addition, it interacts with Ihh to promote the enhancement of ALP activity of osteoblasts and induce the vigorous proliferation of osteoblasts, causing osteosclerosis of the bone tissue.

## Conclusion

The Hh signal plays an important role in fluoride-induced bone injury. The effective inhibition of cyclopamine is expected to be a new target for the treatment of skeletal damage caused by fluorosis.

## Data Availability

We declared that materials described in the manuscript, including all relevant raw data, will be freely available to any scientist wishing to use them for non-commercial purposes, without breaching participant confidentiality.

## Abbreviations

Hh: Hedgehog
SD: Sprague-Dawley
NaF: Sodium fluoride
ALP: Alkaline phosphatase
Ihh: Indian hedgehog
Smo: Smoothened
Gli: Glioma-associated oncogene homolog
Runx2: Runt-related transcription factor 2
BALP: Bone alkaline phosphatase
SPF: Specific pathogen-free
DMSO: Dimethyl sulfoxide
EDTA: Ethylenediaminetetraacetic acid
H&E: Hematoxylin and eosin
IHC: Immunohistochemistry
PMSF: Phenylmethylsulfonyl fluoride
RIPA: Radio-immunoprecipitation assay
PAGE: Polyacrylamide gel electrophoresis
PVDF: Electro-transferred to a polyvinylidene fluoride
TBS-T: Tris-buffered saline/Tween
BSA: Bovine serum albumin
LSD: Least significant difference
Ptch: Patched
OPG/RANKL: Osteoprotegerin/receptor activator of NF-κB ligand
TGF-β: Transforming growth factor-β
Ptc1: Patched-1
